# Antioxidant response is a protective mechanism against nutrient deprivation in *C. elegans*

**DOI:** 10.1038/srep43547

**Published:** 2017-02-23

**Authors:** Jun Tao, Qin-Yi Wu, Yi-Cheng Ma, Yuan-Li Chen, Cheng-Gang Zou

**Affiliations:** 1 State Key Laboratory for Conservation and Utilization of Bio-Resources in Yunnan, Yunnan University, Kunming, Yunnan 650091, China

## Abstract

Animals often experience periods of nutrient deprivation; however, the molecular mechanisms by which animals survive starvation remain largely unknown. In the nematode *Caenorhabditis elegans*, the nuclear receptor DAF-12 acts as a dietary and environmental sensor to orchestrate diverse aspects of development, metabolism, and reproduction. Recently, we have reported that DAF-12 together with co-repressor DIN-1S is required for starvation tolerance by promoting fat mobilization. In this report, we found that genetic inactivation of the DAF-12 signaling promoted the production of reactive oxygen species (ROS) during starvation. ROS mediated systemic necrosis, thereby inducing organismal death. The DAF-12/DIN-1S complex up-regulated the expression of antioxidant genes during starvation. The antioxidant enzyme GST-4 in turn suppressed ROS formation, thereby conferring worm survival. Our findings highlight the importance of antioxidant response in starvation tolerance and provide a novel insight into multiple organisms survive and adapt to periods of nutrient deprivation.

All animals have evolved abilities to improve the chances of survival and reproduction. Among these stress, starvation is one of common stressful situations in nature. To cope with nutrient deprivation, the free-living nematode *Caenorhabditis elegans* can make physiological changes to developmentally arrest at multiple stages, such as L1 diapause^1^, dauer diapause^2^, and adult reproductive diapause^3^. Besides reproductive arrest, L4 or young adult worms survive starvation by promoting fat mobilization, which is mediated by a variety of lipases^4^,^5^. For example, starvation induces the expression of lipase gens *fil-1* and *fil-2* that involved in converting fat storage into energy, and thus maintain whole-body energy homeostasis^4^. Meanwhile, upon fasting, the expression of the lysosomal lipase genes, such as *lipl-1* and *lipl-3*, is up-regulated by a transcription factor HLH-30^5^. These lysosomal lipases induce lipid hydrolysis through lipophagy.

In *C. elegans*, the nuclear receptor DAF-12 orchestrates a switch from arrest to developmental progression in response to environmental and dietary cues, and has been implicated as a signal connecting nutrition, development, and longevity^6^,^7^. By binding with its steroidal ligands, dafachronic acids (DAs), DAF-12 induces reproductive development under favorable conditions such as an abundant food supply, whereas DAF-12 together with co-repressor DIN-1S promotes dauer diapause under harsh environmental conditions such as limited food and overcrowding^6^,^7^. Our recent study has revealed that upon fasting, DAF-12/DIN-1S induces the expression of *tbh-1* that encodes tyramine β-hydroxylase, a key enzyme for octopamine biosynthesis in *C. elegans*. Octopamine up-regulates the expression of the lipase gene *lips-6* in the intestine^8^. LIPS-6, in turn, promotes lipid mobilization to confer starvation resistance.

We noted that the survival rates of *daf-12* and *din-1* mutants were significantly lower than that of *tbh-1* mutants. It is possible that in addition to the octopamine pathway, there is another pathway through which DAF-12/DIN-1S acts to regulate starvation resistance. A previous microarray analysis has revealed that expression of antioxidant and detoxification genes is up-regulated during starvation in *C. elegans*^4^. The fact that DAF-12/DIN-1S mediates resistance to heat and oxidative stress^9^ raises a possibility that the complex probably regulates an antioxidant response during starvation. In this study, we demonstrated that loss of function mutations in *daf-12(rh61rh411)* or *din-1(dh127)* resulted in an increase in reactive oxygen species (ROS) formation, which was involved in worm death, after starvation.

## Results

### DAF-12/DIN-1S is dominant during starvation

**Figure 1:**
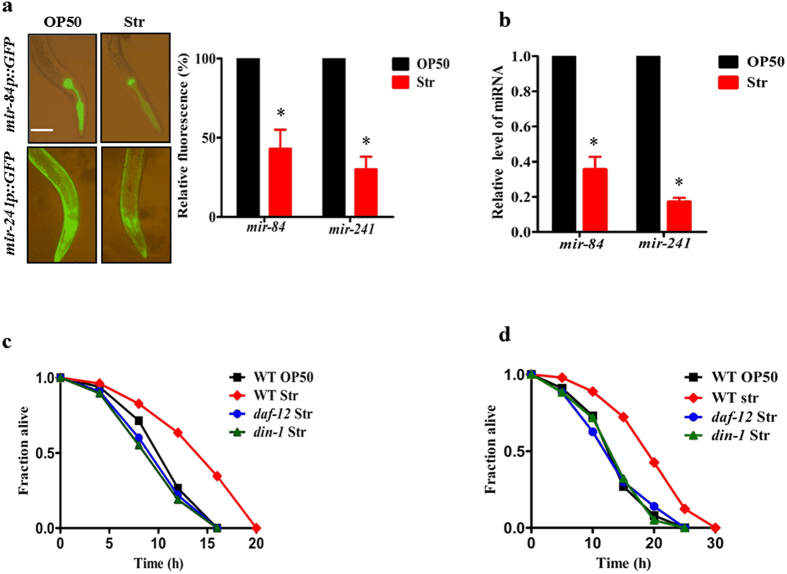
Unliganded DAF-12 is dominant in starved worms. (**a**) The expression of *mir-84p::GFP* and *mir-241p::GFP* in animals was reduced after 12 h of starvation. Quantification of fluorescence intensity is shown in right panel. *P < 0.05 relative to well-fed worms (OP50). Scale bar, 50 μm. (**b**) The expression of *mir-84* and *mir-241* was measured by qPCR. **P < 0.01 relative to well-fed worms. (**c**) The starved wild type (WT) worms were more resistant to menadione (10 mM) than well-fed WT worms (*P* < 0.01). (**d**) The starved WT worms were more resistant to high temperature (35 °C) than well-fed WT worms (*P* < 0.01). Mutations in *daf-12* or *din-1* abolished the resistance to oxidative and heat stress in the starved worms. Str, starvation.

It is believed that DAF-12 is mostly unliganded under starvation conditions, whereas production of DAs by DAF-9 converts DAF-12 to a liganded state when food is available^6^,^10^. To confirm this point, we first monitored the transcriptional activity of liganded DAF-12 using transgenic worms containing *mir-84p::GFP* and *mir-241p::GFP*. Both the microRNAs are the targets of liganded DAF-12, and their expressions were downregulated by mutations in *daf-12* and *daf-9*^11^. As expected, the expression of *mir-84p::GFP* and *mir-241p::GFP* was significantly lower in worms after 12 h starvation than in worms fed with the standard laboratory food *E. coli* OP50 (Fig. 1a). Similar results were obtained by determining the expression of *mir-84* and *mir-241* by qPCR (Fig. 1b). DA-deficient animals (eg. *daf-9* mutant worms) are more stress resistant in a manner dependent on unliganded DAF-12 (the DAF-12/DIN-1S complex)^9^. Consistent with the idea, we found that starved worms exhibited more resistance to a pro-oxidant menadione (10 mM), or high temperature (35 °C), than well-fed worms (Fig. 1c and d). Mutations in *daf-12(rh611rh411)* or *din-1(dh127)* abolished the stress resistance-phenotype of starved worms. These results further confirm this notion that the DAF-12/DIN-1S complex is dominant during nutrient deprivation.

### Starvation induces systemic necrosis in *daf-12(rh61rh411), din-1(dh127),* and *tbh-1(n3247)* mutants

**Figure 2:**
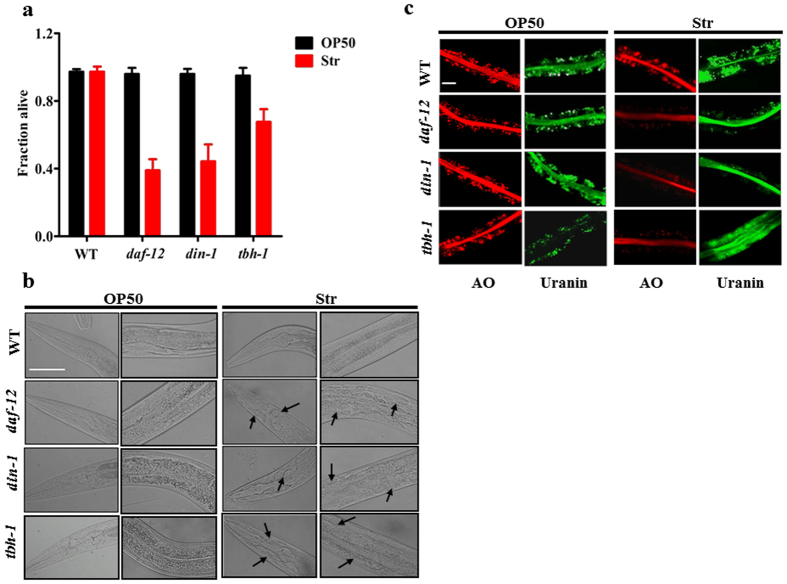
Systemic necrosis occurs in *daf-12, din-1,* and *tbh-1* mutants under nutrient-deprivation conditions. (**a**) Survival rates of worms after five days of starvation. Results are means ± SD of three experiments.*P < 0.05, and **P < 0.01 versus well-fed worms (OP50). WT, wild type worms. (**b** and **c**) Necrosis in worms after two days of starvation. DIC images of worms (**b**) and fluorescence microscopy of acridine orange (AO)-, and uranine- (lower panels) labeled intestine (**c**) are shown. Enlarged vacuoles are indicated by black arrows. Scale bar, 50 μm. Str, starvation.

Our recent study has revealed that the DAF-12/DIN-1S complex induces *tbh-1* expression in response to starvation in *C. elegans*. The DAF-12/DIN-1S-TBH-1 -octopamine signaling is required for starvation tolerance. However, more than 60% of *daf-12(rh61rh411)* or *din-1(dh127)* mutants were dead (Fig. 2a), whereas only 32% of *tbh-1(n3247)* worms were dead after 5 days of starvation. These results suggest that in addition to octopamine, other mechanisms exist for DAF-12/DIN-1S to exert its effect on starvation resistance.

In *C. elegans*, nutrient deprivation induces apoptosis in germ cells and necrotic cell death in neurons^12^,^13^. Thus, organismal death is probably the result of apoptosis or necrosis after prolonged starvation in *daf-12(rh61rh411)* or *din-1(dh127)* mutants. Using the SYTO 12 dye staining against the apoptotic germ cells^14^, we found no apparent accumulation of apoptotic germ cells in the *daf-12(rh61rh411), din-1(dh127)*, and WT worms after two days of starvation (Fig. S1a). Meanwhile, knockdown of *ced-4*, a homolog to mammalian Apaf-1 that is required for apoptosis activation, did not significantly suppress starvation-induced mortality in *daf-12(rh61rh411)* and *din-1(dh127)* mutants after five days of starvation (Fig. S1b). Therefore, our results suggest that worm death from nutrient deprivation does not depend on the core apoptotic machinery.

Next, we examined whether necrosis as a post-starvation phenotype leads to worm death. Differential interference contrast (DIC) images showed that *daf-12(rh61rh411), din-1(dh127)*, and *tbh-1(n3247)* mutants displayed an enlarged vacuolar morphology characteristic of necrotic cells in the head and intestine, whereas WT worms had fewer vacuolated cells after two days of starvation (Fig. 2b). Furthermore, we tested the lysosomal injury using acridine orange, an acidophilic dye that stains lysosomes^15^. We found that acridine orange-labeled granules were lysed in the intestine in *daf-12(rh61rh411), din-1(dh127),* and *tbh-1(n3247)* mutants, but not in WT worms, after two days of starvation (Fig. 2c). Similar results were obtained from staining analysis by the dye uranin, which is an indicator for loss of membrane integrity in lysosome-related organelles^16^ (Fig. 2c). These results indicate that systemic necrosis occurs during starvation.

### Systemic necrosis mediates worm death induced by starvation in *daf-12(rh61rh411)* or *din-1(dh127)* mutants

**Figure 3:**
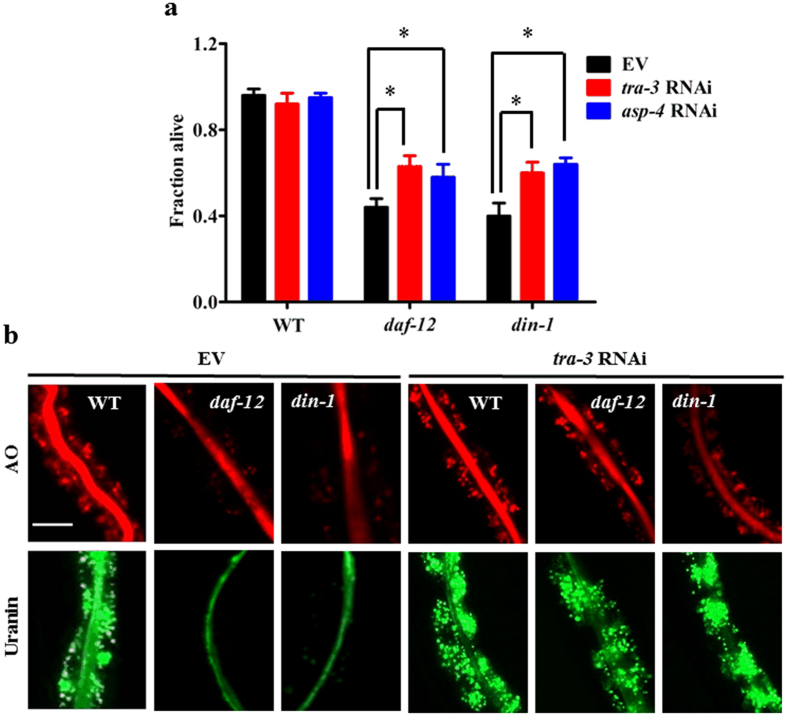
Worm death is due to systemic necrosis under nutrient-deprivation conditions. (**a**) Knockdown of *asp-4* and *tra-3* by RNAi increased the survival in *daf-12(rh61rh411)* or *din-1(dh127)* mutants after five days of starvation. *P < 0.05 versus EV. (**b**) Knockdown of *tra-3* partially inhibited necrosis induced by starvation in *daf-12(rh61rh411)* or *din-1(dh127)* mutants. EV, empty vector. Scale bar, 50 μm.

In *C. elegans*, many genes involved in necrosis have been identified, including calpains and aspartyl proteases^17^,^18^. We first examined whether calpain proteases affect starvation-induced organismal death by knockdown of *clp-1, clp-2, tra-3*/*clp-5, clp-6, clp-7*. Organismal death was significantly suppressed in *daf-12(rh61rh411)* or *din-1(dh127)* mutants subjected to RNAi with *tra-3*/*clp-5* (Fig. 3a), but not with *clp-1, clp-2, clp-6*, and *clp-7* (Fig. S2), after five days of starvation. Meanwhile, knockdown of *tra-3* by RNAi markedly reduced lysosomal injury in *daf-12(rh61rh411)* or *din-1(dh127)* mutants upon starvation (Fig. 3b). There are at least six aspartyl proteases (*asp-1* to *asp-6*) in *C. elegans*. We found that inhibition of *asp-4* by RNAi, but not other aspartyl proteases (Fig. S3), also showed protection (Fig. 3a). These results suggest that necrosis is responsible for starvation-induced organismal death.

### DAF-12/DIN-1S up-regulates the expression of antioxidant genes, which are required for starvation resistance

**Figure 4:**
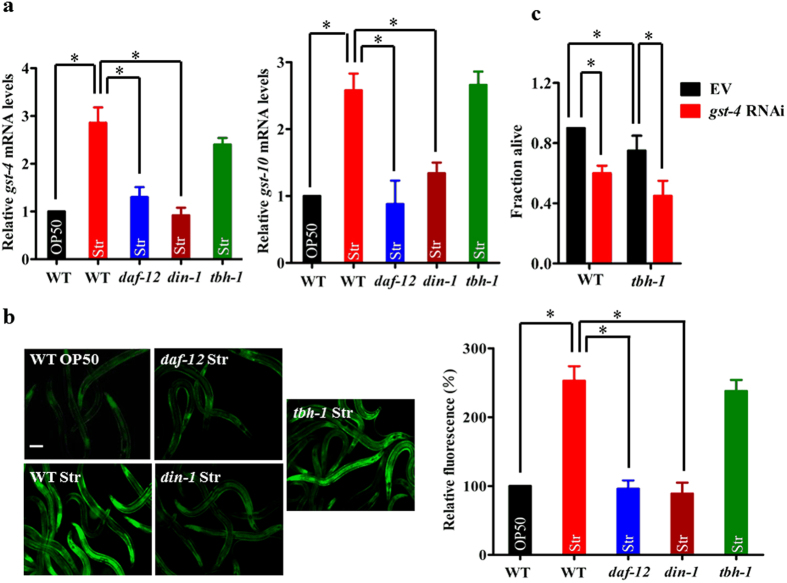
DAF-12/DIN-1S regulates antioxidant genes to promote starvation resistance. (**a**) The expression of *gst-4* (left panel) and *gst-10* (right panel) was significantly up-regulated in WT worms after one day of starvation. Mutations in *daf-12(rh61rh411)* or *din-1(dh127)*, but not *tbh-1(n3247)*, inhibited the expression of *gst-4* and *gst-10* in starved worms. (**b**) The expression of *Pgst-4::GFP* was up-regulated in starved wild type (WT) worms relative to well-fed worms. Mutations in *daf-12(rh61rh411)* or *din-1(dh127)*, but not *tbh-1(n3247)*, inhibited the expression of *Pgst-4::GFP*. The right part shows quantification of GFP levels. (**c**) Knock-down of *gst-4* reduced survival rates after five days of starvation. *P < 0.05. Scale bar, 100 μm. Str, starvation.

It is well established that the DAF-12/DIN-1S complex mediates resistance to heat and oxidative stress^9^. A previous microarray analysis has revealed that a set of stress resistance and detoxification related genes, such as glutathione S-transferases (*gst-4, gst-10, gst-24*, and *gst-26*), UDP-glucuronosyltransferases (*ugt-16, K04A8.10*), and cytochrome P450 (*cyp-35D1* and *cyp-29A3*), is up-regulated during starvation^4^. Among the GSTs, GST-4 and GST-10 play important roles in resistance to oxidative stress in *C. elegans*^19^,^20^. Using qPCR, we found that expression of *gst-4* and *gst-10* was significantly up-regulated in WT and *tbh-1(n3247)* worms, but not in *daf-12(rh61rh411)* or *din-1(dh127)* worms, during one day of starvation (Fig. 4a). Using transgenic worms carrying the *Pgst-4::gst-4::GFP*, we found that the expression of *Pgst-4::gst-4::GFP* was significantly induced by starvation in WT and *tbh-1(n3247)* worms, but not in *daf-12(rh61rh411)* or *din-1(dh127)* worms (Fig. 4b). Thus, the DAF-12/DIN-1S complex, rather than TBH-1, induces the expression of antioxidant genes during starvation. Interestingly, knockdown of *gst-4* by RNAi reduced worm survival in both WT worms and *tbh-1(n3247)* mutants (Fig. 4c). These results suggest that DAF-12/DIN-1S promotes worm survival during starvation, in part, by up-regulating the expression of antioxidant genes.

### ROS are involved in worm death in *daf-12(rh61rh411)* or *din-1(dh127)* worms during starvation

**Figure 5:**
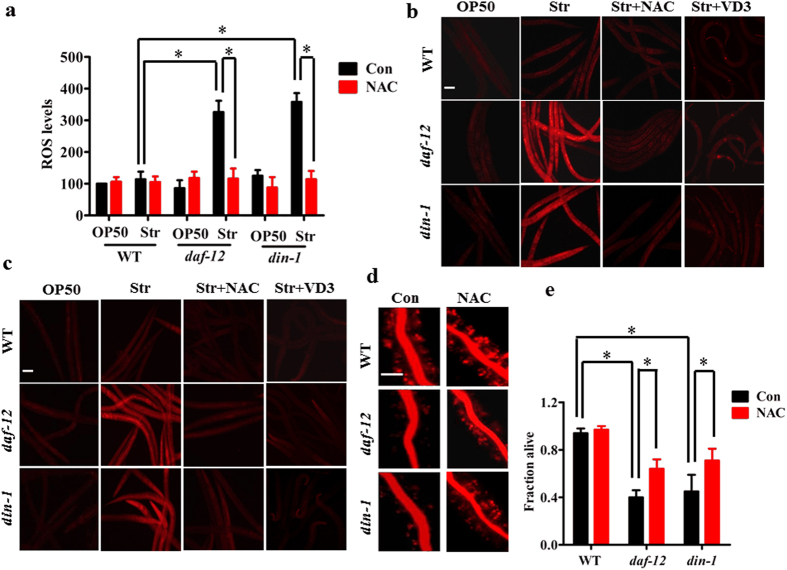
ROS formation is involved in worm death in *daf-12(rh61rh411)* or *din-1(dh127)* worms after starvation. (**a–c**) Mutations in *daf-12(rh61rh411)* or *din-1(dh127)* resulted in an increase in ROS formation after 24 h of starvation. The levels of ROS were detected by DCF (**a**), DHE (**b**), and CellROX^®^ Deep Red (**c**). The antioxidant NAC (1 mM) and vitamin D3 (0.5 mM) markedly diminished the increased ROS levels in starved *daf-12(rh61rh411)* or *din-1(dh127)* mutants. Scale bar, 100 μm. (**d**) NAC significantly suppressed necrosis in the *daf-12(rh61rh411)* or *din-1(dh127)* mutants after starvation. Fluorescence microscopy of acridine orange (AO)-labeled intestine of worms after two days of starvation. Scale bar, 50 μm. (**e**) NAC markedly promoted the survival of *daf-12(rh61rh411)* or *din-1(dh127)* mutants after five days of starvation. Results are means ± SD of three experiments. *P < 0.05. Str, starvation. Con, control.

The induction of antioxidant gene expression during starvation raises a possibility that starvation is able to induce the generation of reactive oxygen species (ROS). To test this idea, we first determined the levels of ROS using 2′,7′-dichlorodihydrofluorescein diacetate (H_2_DCFDA), a fluorescent dye that has been used to detect the ROS levels in *C. elegans*. As shown in Fig. 5a, the basal levels of ROS were very low in WT well-fed worms. However, WT worms subjected to 24 h starvation exhibited similar levels of ROS to WT well-fed worms (Fig. 5a). Interestingly, the levels of ROS were dramatically elevated in *daf-12(rh61rh411)* or *din-1(dh127)* mutants following starvation. It should be noted that a mutation in *daf-12(rh61rh411)* and *din-1(dh127)* itself did not lead to an increase in ROS levels under well-fed conditions. Similar results were obtained using fluorescent dyes, dihydroethidium (DHE) and CellROX^®^ Deep Red (Fig. 5b and c). Likewise, knockdown of *gst-4* also promoted ROS formation during 24 h of starvation (Figs S5 and S6). Recently, Mark *et al*.^21^ have reported that vitamin D3 promotes the expression of *gst-4* in worms, which is dependent on SKN-1 and IRE-1. We found that vitamin D3 significantly up-regulated the expression of *gst-4* in both *daf-12(rh61rh411)* and *din-1(dh127)* mutants after 12 h of starvation (Fig. S4). Meanwhile, vitamin D3 also blocked the increase in ROS formation in starved *daf-12(rh61rh411)* and *din-1(dh127)* mutants (Fig. 5b and c). These data suggest that DAF-12/DIN-1S mediates the induction of *gst-4*, thereby inhibiting ROS formation.

To test whether induction of ROS formation plays a role in starvation-induced organismal death in *daf-12(rh61rh411)* or *din-1(dh127)* mutants, worms were treated with N-acetylcysteine (NAC) (1 mM), a ROS scavenger^22^. NAC markedly diminished the increased ROS levels in *daf-12(rh61rh411)* or *din-1(dh127)* mutants after 24 h starvation (Fig. 5a–c). Importantly, NAC not only significantly suppressed lysosomal injury after two days of starvation (Fig. 5d), but also partially rescued the starvation-induced mortality in *daf-12(rh61rh411)* and *din-1(dh127)* mutants (Fig. 5e).

In addition, the other antioxidants, such as glutathione reduced ethyl ester (GSH-MEE), and α-tocopherol, also suppressed the ROS formation in the starved worms subjected to *gst-4* RNAi (Figs S5 and S6). Thus, starvation-induced expression of antioxidant genes is required for starvation stress.

## Discussion

Our studies uncover a surprising role for the antioxidant response in starvation resistance in *C. elegans*. After nutrient deprivation, the liganded DAF-12 (DAF-12/DA) shifts the equilibrium to ligand-free DAF-12/DIN-1S complex. DAF-12/DIN-1S in turn boosts the antioxidant response, which maintains redox homeostasis. Downregulation of this pathway exacerbates oxidative stress, thereby mediating nutrient deprivation-induced organismal death.

Up-regulation of antioxidant and detoxification genes is widely observed in a variety of organisms under nutrient deprivation conditions. A microarray analysis reveals that a set of stress resistance and detoxification related genes is up-regulated during starvation in adult worms^4^. Furthermore, large numbers of antioxidant and detoxification genes encoding glutathione peroxidases, superoxide dismutases, GSTs, and UGTs, are up-regulated in dauer larva of *C. elegans*, which is a non-feeding alternative larval stage^23^,^24^,^25^. In yeast, methionine starvation induces expression of antioxidant genes such as superoxide dismutases, thioredoxin, peroxiredoxin, glutaredoxin^26^. In brown trout, the malondialdehyde levels and the activities of several antioxidant enzymes (eg. superoxide dismutase, catalase, glutathione peroxidase, and glutathione reductase) increased in liver and gills during long-term starvation^27^. These results implicate that starvation is a potential factor that elicits oxidative stress. In this study, the observation that genetic inactivation of *daf-12* or *din-1* significantly elicits ROS formation during starvation supports this idea.

Starvation response protects mice and yeast against oxidative stress^28^,^29^. Meanwhile, constitutive activation of the detoxification/antioxidant response factor SKN-1/Nrf-1 induces a starvation adaptation response in *C. elegans* and mice^30^. In this study, our results indicate that starvation promotes the resistance to oxidative and heat stresses in adult worms, which is dependent on the DAF-12/DIN-1S complex. These results imply that starvation and antioxidant defense are intimately linked with each other. Indeed, a previous study has demonstrated that the antioxidant genes that detoxify ROS are correlated with starvation survival in yeast^26^. We found that DAF-12/DIN-1S controls expression of several antioxidant genes, such as *gst-4* and *gst-10* and. Knockdown of *gst-4* increases the abundance of ROS, and worm death during starvation. More importantly, suppression of ROS by NAC partially rescues worm death in *daf-12(rh61rh411)* or *din-1(dh127)* mutants. These results suggest that the DAF-12/DIN-1S complex confers starvation resistance, at least in part, by a mechanism of its action involved in the antioxidant defense. To counter against elevated ROS-induced tissue injury, compensatory antioxidant response is crucial for starvation tolerance. Clearly, the mechanism underlying the DAF-12/DIN-1S complex-mediated antioxidant response needs to be investigated further in light of our current results.

At present, the mechanisms underlying death of the whole organism after nutrient deprivation remain incompletely understood. Although apoptosis occurs in germ cells of worms after nutrient deprivation^12^,^31^, our results demonstrated that inactivation of the apoptosis gene cascade fails to prevent worm death, excluding a role for apoptosis in this process. We found that starvation triggers systemic necrosis in *daf-12(rh61rh411)* and *din-1(dh127)* mutants. Genetic inactivation of components in the necrosis pathway (calpains and cathepsins) can delay worm death, confirming a role for systemic necrosis in starvation-induced organismal death. Although we tested the effect of five calpains and six aspartyl proteases on worm death, however, only knockdown of *tra-3* and *asp-4* by RNAi inhibits worm death. In this study, we tested the knockdown efficiency of RNAi on knockdown of all of the calpains and aspartyl proteases, and found these genes expressions were significantly ablated by RNAi. However, we really do not know why only knockdown of *tra-3* and *asp-4* displays a significant protection. As the antioxidant NAC significantly suppresses systemic necrosis in *daf-12(rh61rh411)* and *din-1(dh127)* mutants during starvation, ROS is likely to be involved in systemic necrosis.

In summary, our results demonstrate in *C. elegans* that DAF-12/DIN-1S, which is activated during starvation, up-regulates antioxidant genes. Induction of antioxidant responses clears the detrimental effects of ROS on worm tissues, and ultimately promotes survival during starvation. Increased antioxidant response seems to be a common phenomenon among many organisms during nutrient deprivation. Thus, dysregulation of antioxidant response contributes to cell death in the process.

## Materials and Methods

### Nematode strains

The *C. elegans* strains were cultured under standard conditions and fed *E. coli* OP50^32^. Wild-type animals were *C. elegans* Bristol N2. Mutated strains used in this stud, including *daf-12(rh61rh411), din-1(dh127), tbh-1(n3247)*, and strains containing *mir-84p::GFP, mir-241p::GFP*, and *Pgst-4::gst-4::GFP*, were kindly provided by the Caenorhabditis Genetics Center (CGC), which is funded by NIH Office of Research Infrastructure Programs (P40 OD010440).

### RNA interference

The clones of genes for RNAi were from the Ahringer library^33^. All RNAi was induced by feeding on synchronized L1 larvae at 20 °C. These worms were cultivated at 20 °C until the young adult stage. Then young adult worms were transferred to the NGM plates for further assays.

### Oxidative and heat stress experiments

After synchronized young adult worms were starved for 12 h, the worms were transferred to plates containing 10 mM menadione (Sigma, St. Louis, MO) in the absence of standard food *Escherichia coli* OP50. Well-fed young adult worms in plates containing 10 mM menadione, and *E. coli* OP50 were used as control. For heat stress, 40–50 young adult worms were transferred to plates in the absence of *E. coli* OP50 at 35 °C. Well-fed young adult worms in the presence of *E. coli* OP50 were used as control. The number of living worms was counted at 5 h intervals until all of the worms were dead. Immobile worms unresponsive to touch were scored as dead. Three plates were performed per assay and all experiments were performed three times.

### Starvation survival analysis

Synchronized populations of L1 larva were cultivated on NGM plates in the presence of *E. coli* OP50 at 20 °C until the young adult stage. 40–50 worms were then transferred to NGM agar plates containing 5′-fluoro- 2′-deoxyuridine (FUdR) (75 μg/ml), ampicillin (100 μg/ml), kanamycin (50 μg/ml), and amphotericin-B (0.25 μg/ml) in the presence or absence of *E. coli* OP50 at 20 °C. The number of living worms was counted at five days. Immobile worms unresponsive to touch were scored as dead. Three plates were performed per assay and all experiments were performed three times.

### Detection of apoptosis

After worms were starved for two days, the worms were staining with M9 medium containing 20 μM SYTO 12 green (Invitrogen) for 90 min^14^. Then the worms were mounted in M9 onto microscope slides. The green fluorescence was monitored using a Nikon e800 fluorescence microscope (Nikon, Tokyo, Japan). At least 25 worms were examined under each condition in three independent experiments.

### Analysis for necrosis

After worms were starved for two days, the worms were stained in M9 medium containing 1 mM acridine orange (Sangon Biotech Co., Shanghai, China)^15^, or 20 mg/ml uranine (Sangon)^16^ for 2 h. After washing with M9 medium for three times, the worms were mounted in M9 onto microscope slides. The red fluorescence of acridine orange and the green fluorescence of uranine were monitored using a Nikon e800 fluorescence microscope. At least 25 worms were examined under each condition in three independent experiments.

### Fluorescence microscopic analysis of GFP-labeled worms

For *mir-84p::GFP, mir-241p::GFP*, and *Pgst-4::gst-4::GFP* analysis, synchronized young adult worms were starved for 12 h. Then the worms were mounted in M9 onto microscope slides. The slides were imaged using a Nikon e800 fluorescence microscope. Fluorescence intensity was quantified by using the ImageJ software (NIH). Mean value and standard errors were calculated based on more than 100 worms under each condition in three independent experiments.

### Quantitative RT-PCR

Total RNA was isolated from worms with TRIzol Reagent (Invitrogen). Random-primed cDNAs were generated by reverse transcription of the total RNA samples with SuperScript II (Invitrogen). A real time-PCR analysis was conducted using SYBR^®^ Premix-Ex TagTM (Takara, Dalian, China) on a Roche LightCycler 480^®^ System (Roche Applied Science, Penzberg, Germany). The primers used for PCR were as follows: *gst-4*: 5′-TGG AGA CTC ATT GAC TTG GG-3′ (F), 5′-TCC TTT CTT GTT GCC ACG-3′ (R); *gst-10*: 5′-CGT GCC ACA ACT TTA CTA CTT C-3′ (F), 5′-CAA CTG ACC AAG GAG CAT TC-3′(R); *act-1*: 5′-GGG CGA AGA AGG AAA TGG TC-3′ (F), 5′-CAG GTG GCG TAG GTG GAG AA-3′ (R).

### Measurement of ROS

After starvation for 24 h, the ROS levels were detected by 2′,7′-dichlorodihydrofluorescein diacetate (DCF-DA) as a probe as described previously^34^. Meanwhile, ROS formation was also detected using two fluorescent dyes dihydroethidium (DHE) and CellROX^®^ Deep Red Reagent, respectively. Briefly, after 24 h of starvation, worms were incubated with 3 μM of DHE in M9 medium for 30 min. Then worms were washed three with PBS, and mounted in M9 onto microscope slides. For ROS detection using CellROX^®^ Deep Red Reagent, worms were fixed by 2% of paraformaldehyde for 30 min. After washed with M9 medium for three times, the worms were incubated with 5 μM of CellROX^®^ Deep Red Reagent for 1 h. Then worms were washed three with PBS, and mounted in M9 onto microscope slides. The slides were imaged using a Nikon e800 fluorescence microscope. Mean value and standard errors were calculated based on more than 100 worms under each condition in three independent experiments.

### Statistics

The statistical significance of differences in gene expression, starvation survival, and fluorescence intensity was assessed by performing a one-way ANOVA followed by a Student-Newman-Keuls test. Differences in survival rates for oxidative/ heat stress treatment were analyzed using the log-rank test. Data were analyzed using SPSS17.0 software (SPSS Inc.).

## Additional Information

**How to cite this article:** Tao, J. *et al*. Antioxidant response is a protective mechanism against nutrient deprivation in *C. elegans. Sci. Rep.* **7**, 43547; doi: 10.1038/srep43547 (2017).

**Publisher's note:** Springer Nature remains neutral with regard to jurisdictional claims in published maps and institutional affiliations.

## Supplementary Material

Supplementary Figures
