# Supplementary material for: Antioxidant response is a protective mechanism against nutrient deprivation in C. elegans
Source: Sci Rep. 2017 Feb 23;7:43547. doi: 10.1038/srep43547 (PMC5322524; doi:10.1038/srep43547)
Supplement: Supplementary Figures [file srep43547-s1.pdf]

Antioxidant response is a protective mechanism against nutrient deprivation in *C. elegans*

Jun Tao, Qin-Yi Wu, Yi-Cheng Ma, Yuan-Li Chen, and Cheng-Gang Zou

Supplemental Figures

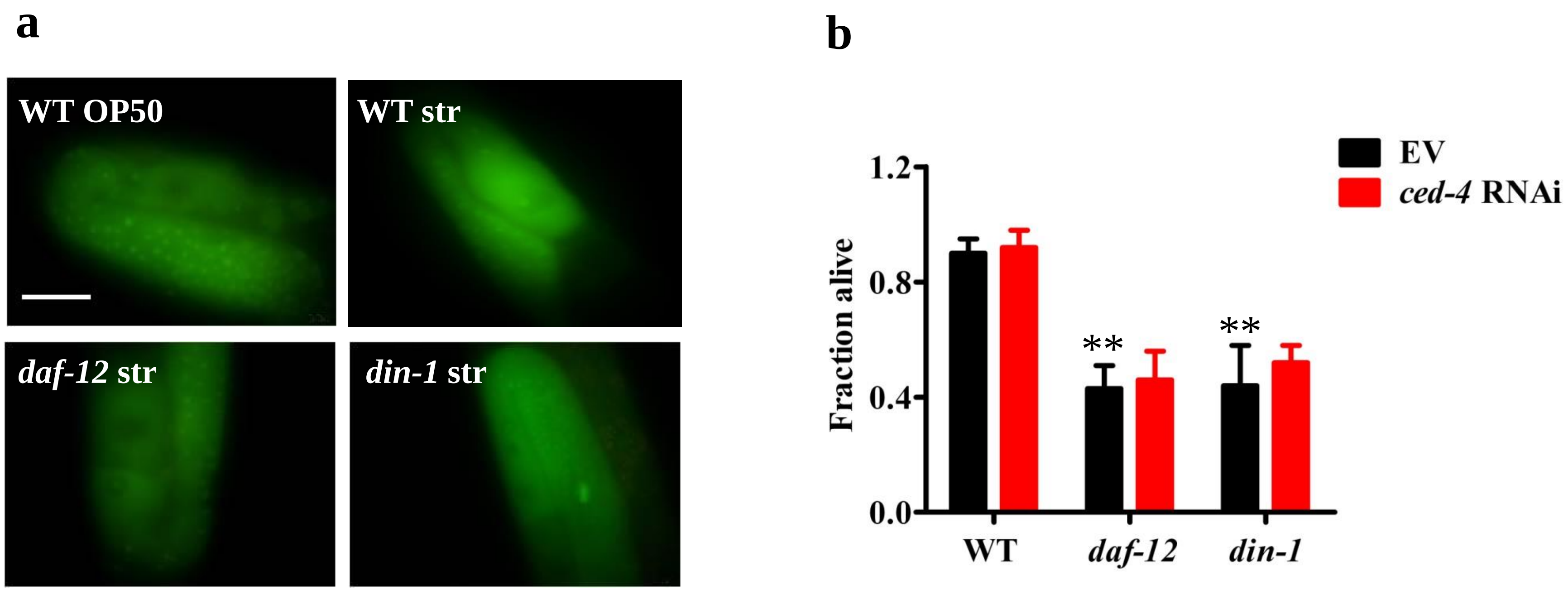

Figure S1 Apoptosis is not involved in worm death after starvation.

(a) SYTO12-stained gonads of *daf-12(rh61rh411)*, *din-1(dh127)*, and WT worms after two days of starvation. (b) Knockdown of *ced-4* did not influenced starvation-induced mortality in *daf-12(rh61rh411)* and *din-1(dh127)* mutants after five days of starvation. Results are means ± SD of three experiments. ***P* < 0.01 relative to WT worms. Scale bar, 20 μm. Str, starvation.

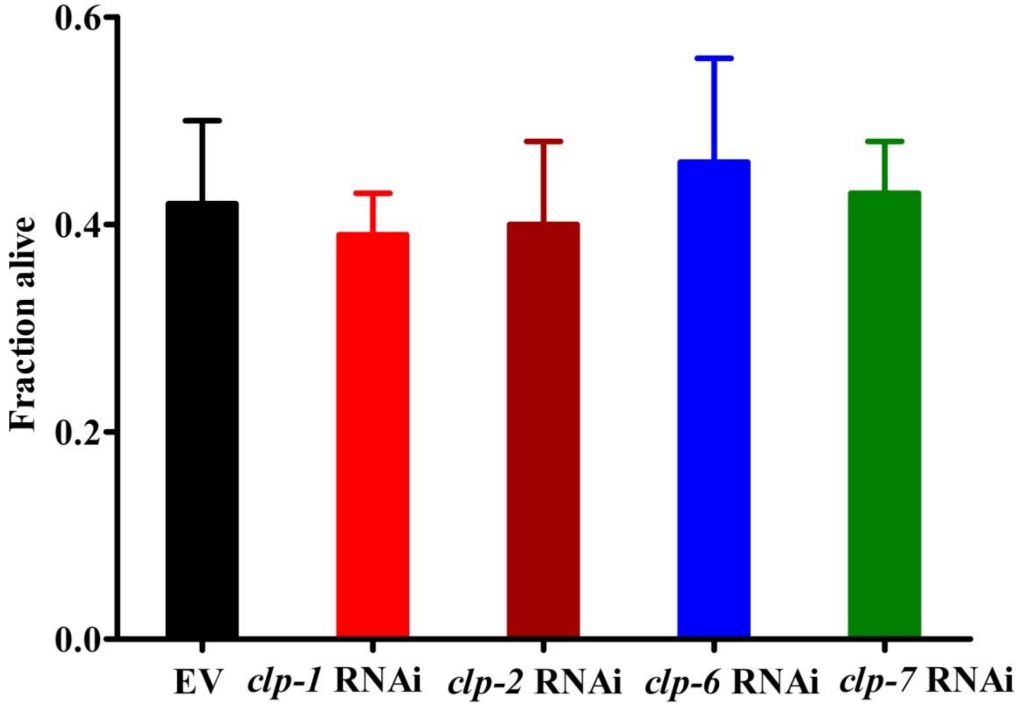

Figure S2. Knockdown of *clp-1*, *clp-2*, *clp-6*, and *clp-7* fails to inhibit death in *daf-12(rh61rh411)* after five days of starvation. EV, empty vector.

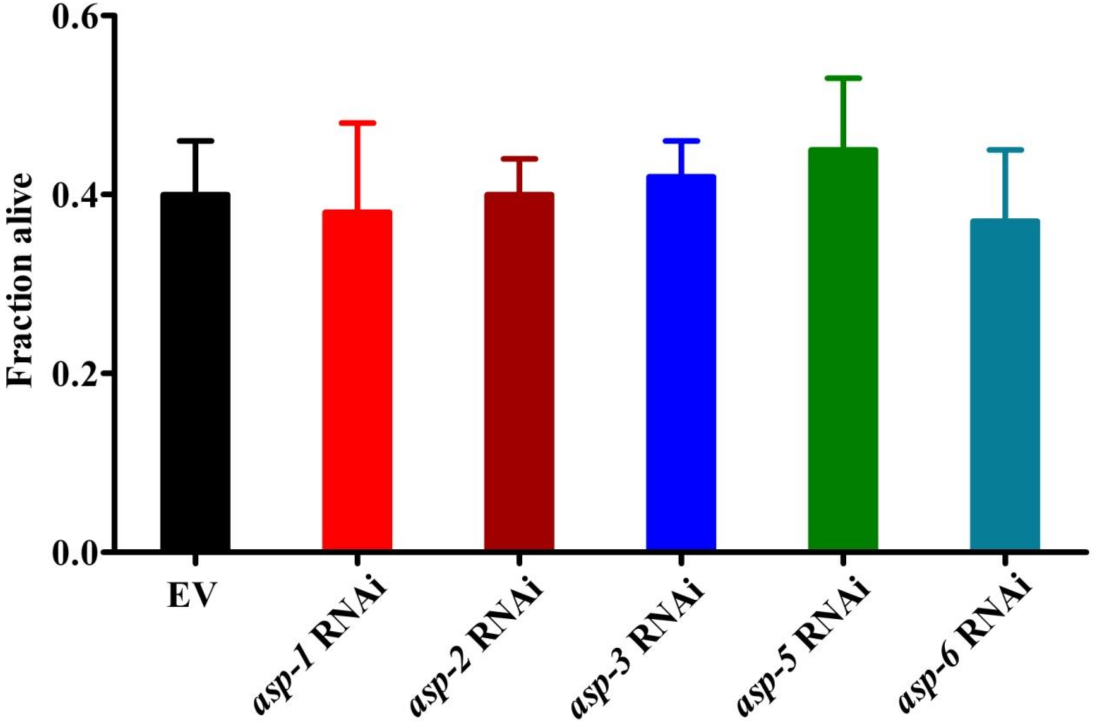

Figure S3. Knockdown of *asp-1*, *asp-2*, *asp-3*, *asp-5*, and *asp-6* fails to inhibit death in *daf-12(rh61rh411)* after five days of starvation. EV, empty vector.

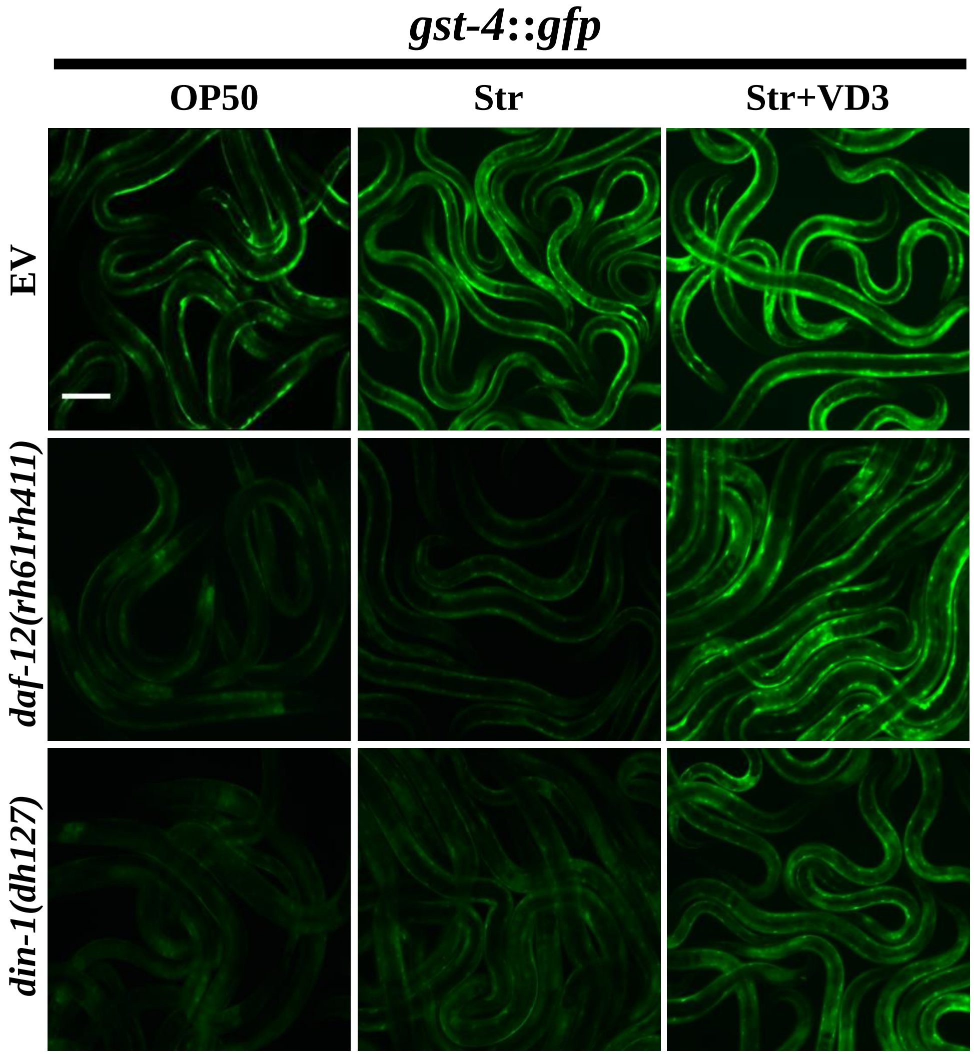

Figure S4. Vitamin D3 promotes the expression of *gst-4* in *daf-12(rh61rh411)* and *din-1(dh127)* mutants after 12h of starvation. Mutations in *daf-12(rh61rh411)* or *din-1(dh127)* inhibited the expression of *Pgst-4::GFP*. Addition of vitamin D3 (0.5 mM) restored the expression of *Pgst-4::GFP*. EV, empty vector; Str, starvation. Scale bar, 100 μm.

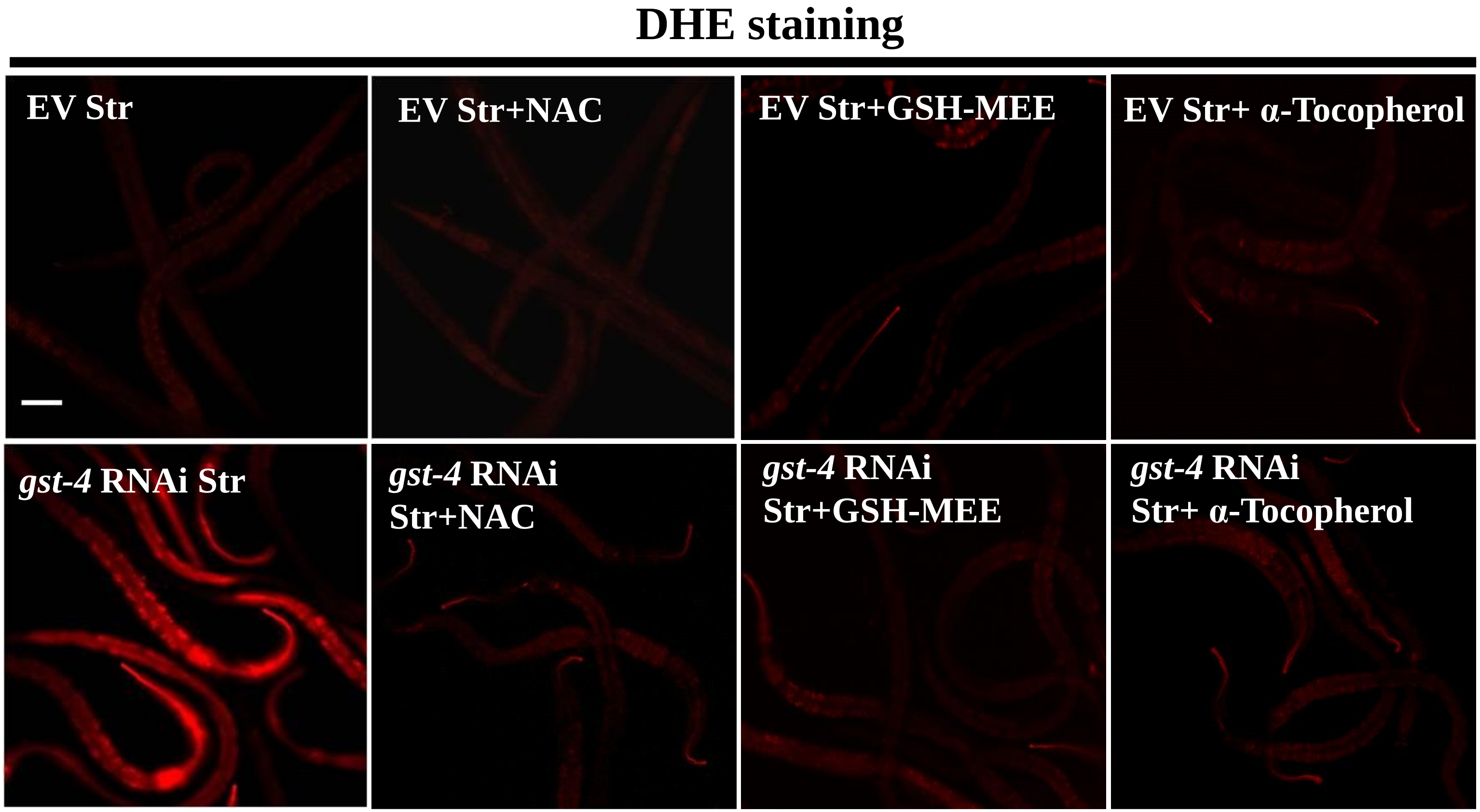

Figure S5. Knockdown of *gst-4* by RNAi significantly increased ROS formation detected by DHE staining. However, antioxidants, NAC (1 mM), GSH-MEE (1 mM), and α-tocopherol (1 mM), markedly diminished the increased ROS levels, measured after one day of starvation. Scale bar, 100 μm. EV, empty vector; Str, starvation.

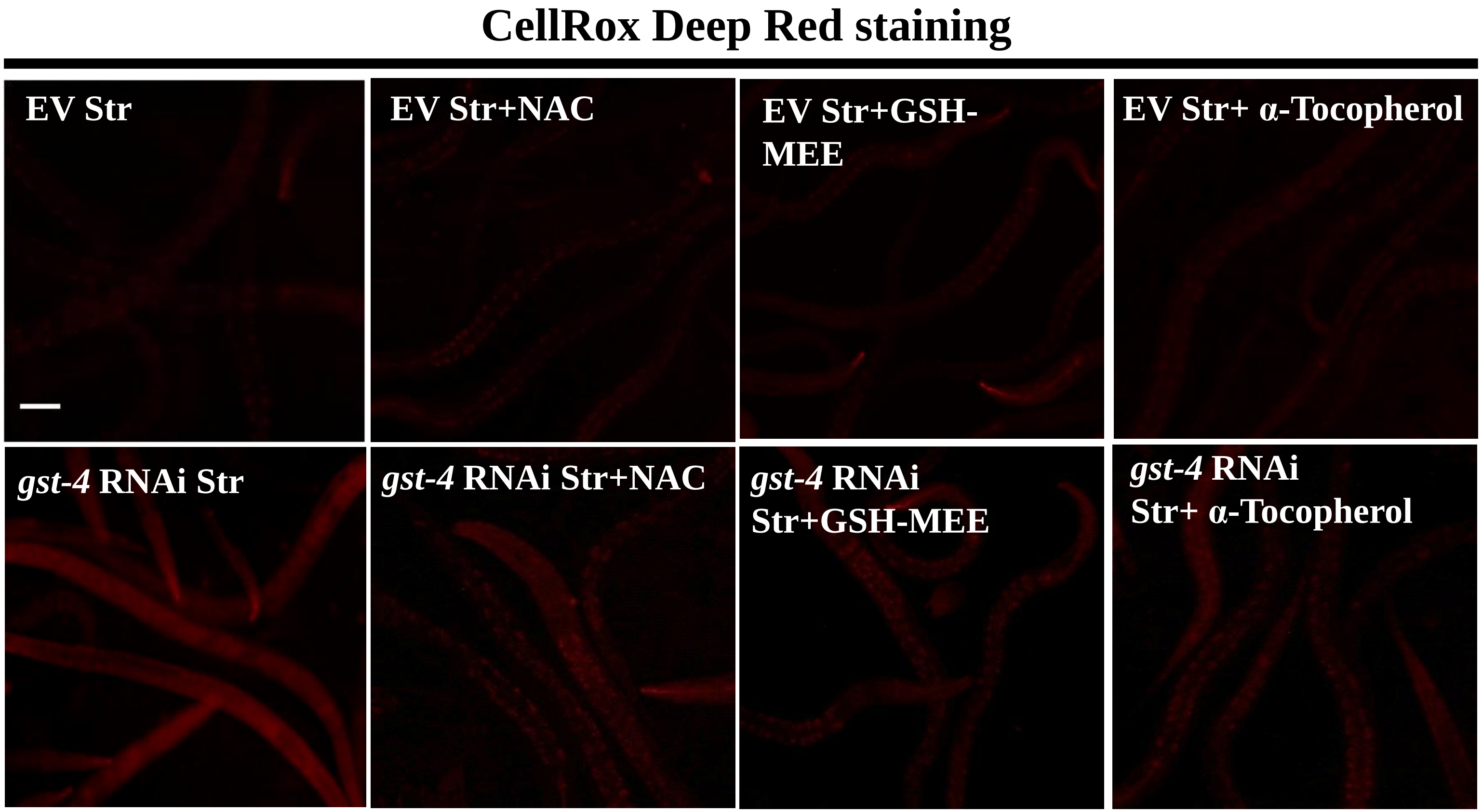

Figure S6. Knockdown of *gst-4* by RNAi significantly increased ROS formation detected by CellRox Deep Red staining. However, antioxidants, NAC (1 mM), GSH-MEE (1 mM), and α-tocopherol (1 mM), markedly diminished the increased ROS levels in worms after one day of starvation. Scale bar, 100 μm. EV, empty vector; Str, starvation.
